# MetaCRAM: an integrated pipeline for metagenomic taxonomy identification and compression

**DOI:** 10.1186/s12859-016-0932-x

**Published:** 2016-02-19

**Authors:** Minji Kim, Xiejia Zhang, Jonathan G. Ligo, Farzad Farnoud, Venugopal V. Veeravalli, Olgica Milenkovic

**Affiliations:** Department of Electrical and Computer Engineering, University of Illinois at Urbana-Champaign, Urbana, 61801 USA; Department of Electrical Engineering, California Institute of Technology, Pasadena, 91125 USA

**Keywords:** Metagenomics, Genomic compression, Parallel algorithms

## Abstract

**Background:**

Metagenomics is a genomics research discipline devoted to the study of microbial communities in environmental samples and human and animal organs and tissues. Sequenced metagenomic samples usually comprise reads from a large number of different bacterial communities and hence tend to result in large file sizes, typically ranging between 1–10 GB. This leads to challenges in analyzing, transferring and storing metagenomic data. In order to overcome these data processing issues, we introduce MetaCRAM, the first *de novo*, parallelized software suite specialized for FASTA and FASTQ format metagenomic read processing and lossless compression.

**Results:**

MetaCRAM integrates algorithms for taxonomy identification and assembly, and introduces parallel execution methods; furthermore, it enables genome reference selection and CRAM based compression. MetaCRAM also uses novel reference-based compression methods designed through extensive studies of integer compression techniques and through fitting of empirical distributions of metagenomic read-reference positions. MetaCRAM is a lossless method compatible with standard CRAM formats, and it allows for fast selection of relevant files in the compressed domain via maintenance of taxonomy information. The performance of MetaCRAM as a stand-alone compression platform was evaluated on various metagenomic samples from the NCBI Sequence Read Archive, suggesting 2- to 4-fold compression ratio improvements compared to gzip. On average, the compressed file sizes were 2-13 percent of the original raw metagenomic file sizes.

**Conclusions:**

We described the first architecture for reference-based, lossless compression of metagenomic data. The compression scheme proposed offers significantly improved compression ratios as compared to off-the-shelf methods such as zip programs. Furthermore, it enables running different components in parallel and it provides the user with taxonomic and assembly information generated during execution of the compression pipeline.

**Availability:**

The MetaCRAM software is freely available at http://web.engr.illinois.edu/~mkim158/metacram.html. The website also contains a README file and other relevant instructions for running the code. Note that to run the code one needs a minimum of 16 GB of RAM. In addition, virtual box is set up on a 4GB RAM machine for users to run a simple demonstration.

**Electronic supplementary material:**

The online version of this article (doi:10.1186/s12859-016-0932-x) contains supplementary material, which is available to authorized users.

## Background

Metagenomics is an emerging discipline focused on genomic studies of complex microorganismal population. In particular, metagenomics enables a range of analyses pertaining to species composition, the properties of the species and their genes as well as their influence on the host organism or the environment. As the interactions between microbial populations and their hosts plays an important role in the development and functionality of the host, metagenomics is becoming an increasingly important research area in biology, environmental and medical sciences. As an example, the National Institute of Health (NIH) recently initiated a far-reaching Human Microbiome Project [1] which has the aim to identify species living at different sites of the human body (in particular, the gut and skin [2]), observe their roles in regulating metabolism and digestion, and evaluate their influence on the immune system. The findings of such studies may have important impacts on our understanding of the influence of microbials on an individual’s health and disease, and hence aid in developing personalized medicine approaches. Another example is the Sorcerer II Global Ocean Sampling Expedition [3], led by the Craig Venter Institute, the purpose of which is to study microorganisms that live in the ocean and influence/maintain the fragile equilibrium of this ecosystem.

There are many challenges in metagenomic data analysis. Unlike classical genomic samples, metagenomic samples comprise many diverse organisms, the majority of which is usually unknown. Furthermore, due to low sequencing depth, most widely used assembly methods – in particular, those based on de Bruijn graphs – often fail to produce quality results and it remains a challenge to develop accurate and sensitive meta-assemblers. These and other issues are further exacerbated by the very large file size of the samples and their ever increasing number. Nevertheless, many algorithmic methods have been developed to facilitate some aspects of microbial population analysis: examples include MEGAN (MEta Genome ANalyzer) [4], a widely used tool that allows for an integrative analysis of metagenomic, metatranscriptomic, metaproteomic, and rRNA data; and PICRUSt (Phylogenetic Investigation of Communities by Reconstruction of Unobserved States) [5], developed to predict metagenome functional contents from 16S rRNA marker gene sequences. Although suitable for taxonomic and functional analysis of data, neither MEGAN nor PICRUSt involve a data compression component, as is to be expected from highly specialized analytic software.

In parallel, a wide range of software solutions have been developed to efficiently compress classical genomic data (a comprehensive survey of the state-of-the-art techniques may be found in [6]). Specialized methods for compressing whole genomes have been reported in [7–9], building upon methods such as modified Lempel-Ziv encoding and the Burrows-Wheeler transform. Compression of reads is achieved by mapping the reads to reference genomes and encoding only the differences between the reference and the read; or, in a *de novo* fashion that does not rely on references and uses classical sequence compression methods. Quip [10] and CRAM [11] are two of the best known reference-based compression algorithms, whereas ReCoil [12], SCALCE [13], MFCompress [14], and the NCBI Sequence Read Archive method compress data without the use of reference genomes. Reference-based algorithms in general achieve better compression ratios than reference-free algorithms by exploiting the similarity between some predetermined reference and the newly sequenced reads. Unfortunately, none of the current reference-based method can be successfully applied to metagenomic data, due to the inherent lack of “good” or known reference genomes. Hence, the only means for compressing metagenomic FASTA and FASTQ files is through the use of *de novo* compression methods.

As a solution to the metagenomic big data problem, we introduce MetaCRAM, the first *de novo*, parallel, CRAM-like software specialized for FASTA-format metagenomic read compression, which in addition provides taxonomy identification, alignment and assembly information. This information primarily facilitates compression, but also allows for fast searching of the data in the compressive domain and for basic metagenomic analysis. The gist of the classification method is to use a taxonomy identification tool – in this case, Kraken [15] – which can accurately identify a sufficiently large number of organisms from a metagenomic mix. By aligning the reads to the identified reference genomes of organisms via Bowtie2 [16], one can perform efficient *lossless* reference-based compression via the CRAM suite. Those reads not aligned to any of the references can be assembled into contigs through existing metagenome assembly software algorithms, such as Velvet [17] or IDBA-UD [18]; sufficiently long contigs can subsequently be used to identify additional references through BLAST (Basic Local Alignment Search Tool) [19]. The reads aligned to references are compressed into the standard CRAM format [11], using three different integer encoding methods, Huffman [20], Golomb [21], and Extended Golomb encoding [22].

MetaCRAM is an automated software with many options that accommodate different user preferences, and it is compatible with the standard CRAM and SAMtools data format. In addition, its default operational mode is lossless, although additional savings are possible if one opts for discarding read ID information. We report on both the lossless and “lossy” techniques in the “Methods” Section. MetaCRAM also separates the read compression process from the quality score compression technique, as the former technique is by now well understood while the latter is subject to constant changes due to different quality score formats in sequencing technologies. These changes may be attributed to increasing qualities of reads and changes in the correlations of the score values which depend on the sequencing platform. For quality score compression, the recommended method is QualComp [23].

MetaCRAM offers significant compression ratio improvements when compared to standard bzip and gzip methods, and methods that directly compress raw reads. These improvements range from 2–4 fold file size reductions, which leads to large storage cost reductions. Furthermore, although MetaCRAM has a relatively long compression phase, decompression may be performed in a matter of minutes. This makes the method suitable for both real time and archival applications.

The paper is organized as follows. The “Results” Section contains an in-depth performance analysis of MetaCRAM with respect to processing and retrieval time, and achievable compression ratios. The “Discussion” Section describes the advantages of using MetaCRAM for data compression compared to other general-purpose methods, and describes directions for future algorithmic improvements. The “Methods” Section contains detailed information about the methodology behind the MetaCRAM algorithmic blocks and it also outlines the way constituent algorithms are integrated and their purposes in the pipeline.

## Results

**Fig. 1:**
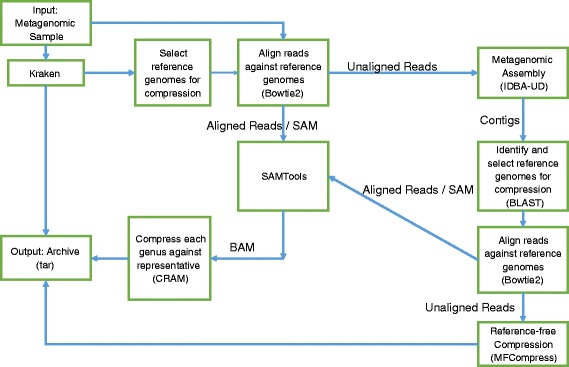
Block Diagram. The block diagram of the MetaCRAM Algorithm for Metagenomic Data Processing and Compression. Its main components are taxonomy identification, alignment, assembly and compression

The block diagram of the MetaCRAM algorithm is given in Fig. 1, and the operation of the algorithm may be succinctly explained as follows. The first step is to identify suitable references for compression, which is achieved by identifying dominant taxonomies in the sample. The number of references is chosen based on cut-off abundance thresholds, which themselves are chosen using several criteria that trade-off compression ratio and compression time. Once the references are chosen, the raw reads are aligned to their closest references and the starting positions of the reads are statistically analyzed to determine the best integer compression method to be used for their encoding. Furthermore, reads that do not align sufficiently well with any of the chosen references are assembled using IDBA_UD, and the contig outputs of the assembler are used to identify additional references via BLAST search. Reads not matched with any references after multiple iterations of the above procedure are compressed independently with the MFCompress suite. The results associated with each of the described processing stages are discussed in the next subsections. Note that here and throughout the paper, we use standard terms in genomics and bioinformatics without explanations.

We tested MetaCRAM as a stand-alone platform and compared it to MFCompress, a recently developed software suite specialized for FASTA files, and bzip2 and gzip [24], standard general purpose compression tools (available at http://www.bzip.org). Other software tools for compression of sequencing data such as SCALCE and Quip, and SAMZIP [25] and SlimGene [26], were not tested because they were either for FASTQ or SAM file formats, and not FASTA files.

As already pointed out, MetaCRAM does not directly process FASTQ file formats for multiple reasons: 1) the quality of sequencers are improving significantly, reaching the point where quality scores may contain very little information actually used during analysis; 2) reads with low quality scores are usually discarded and not included in metagenomics analysis – only high quality sequences are kept; 3) there exist software tools such as QualComp [23], specifically designed for compressing quality scores that users can run independently along with MetaCRAM.

### Taxonomy identification and reference genome selection

As the first step of our analysis, we compared two metagenomic taxonomy identification programs, Kraken and MetaPhyler in terms of computation time and identification accuracy on synthetic data, as it is impossible to test the accuracy of taxonomy identification on real biological datasets. For this purpose, we created mixtures of reads from 15 species, listed in the Additional file 1. The two Illumina paired-end read files were created by MetaSim [27] with 1 % error rate, and they amounted to a file of size 6.7 GB. Kraken finished its processing task in 22 min and successfully identified all species within the top 50 most abundant taxons. On the other hand, MetaPhyler ran for 182 min and failed to identify Acetobacterium woodii and Haloterrigena turkmenica at the *genus* level. This example illustrates a *general trend* in our comparative findings, and we therefore adopted Kraken as a default taxonomy retrieval tool for MetaCRAM.

When deciding how to choose references for compression, one of the key questions is to decide which outputs of the Kraken taxonomy identification tool are relevant. Recall that Kraken reports the species identified according to the number of reads matched to their genomes. The most logical approach to this problem is hence to choose a threshold for the abundance values of reads representing different bacterial species, and only use sequences of species with high abundance as compression references. Unfortunately, the choice for the optimal threshold value is unclear and it may differ from one dataset to another; at the same time, the threshold is a key parameter that determines the overall compression ratio – choosing too few references may lead to poor compression due to the lack of quality alignments, while choosing too many references may reduce the compression ratio due to the existence of many pointers to the reference files. In addition, if we allow too many references, we sacrifice computation time for the same final alignment rate. It is therefore important to test the impact of the threshold choice on the resulting number of selected reference genomes.

In Table 1, we listed our comparison results for all five datasets studied, using two threshold values: 75 (high) and 10 (low). For these two choices, the results are colored gray and white, respectively. We observe that we get slightly worse compression ratios if we select too few references, as may be seen for the files ERR321482 and ERR532393. Still, the processing time is significantly smaller when using fewer references, leading to 30 to 80 minutes of savings in real time. It is worth to point out that this result may also be due to the different qualities of internal hard drives: for example, the columns in gray were obtained running the code on Seagate Barracuda ST3000, while the results listed in white were obtained via testing on Western Digital NAS.

**Table 1:** Analysis of the influence of different threshold values on reference genome selection after taxonomy identification and compression ratios

Data	Original (MB)	**Comp. (MB)**	**Processing time**	**Align. %**	**No. files**	Comp. (MB)	Processing time	Align. %	No. files
ERR321482	1429	191	299 m 20 s	26.99	211	193	239 m 28 s	24.22	29
			422 m 21 s	3.57	1480		398 m 3 s	6.5	1567
			12 m 24 s				8 m 13 s		
SRR359032	3981	319	127 m 34 s	57.72	26	320	93 m 60 s	57.71	7
			245 m 53 s	9.7	30		206 m 18 s	9.71	32
			8 m 37s				7 m 27 s		
ERR532393	8230	948	639 m 55 s	45.78	267	963	522 m	42.45	39
			1061 m 50 s	1.98	1456		1067 m 49 s	7.16	1639
			73 m 59 s				28 m 13s		
SRR1450398	5399	703	440 m 4 s	7.14	190	703	364 m 34 s	6.82	26
			866 m 56 s	0.6	793		790 m 52 s	0.91	818
			21 m 2 s				17 m 38 s		
SRR062462	6478	137	217 m 21 s	2.55	278	139	197 m 15 s	2.13	50
			254 m 26 s	0.13	570		241 m 2 s	0.51	656
			15 m 45 s				19 m 31 s		

Columns in bold represent a threshold of 75 species, while the columns not bolded correspond to a cutoff of 10 species. The results are shown for MetaCRAM-Huffman. “Align. %” refers to the alignment rates for the first and second round, and “No. files” refers to the number of reference genome files selected in the first and second iteration. Processing times are recorded row by row denoting real, user, and system time in order

Many of the most abundant references may be from the same genus, and this may potentially lead to the problem of multiple alignment due to subspecies redundancy. The almost negligible effect of the number of reference genomes on alignment rate implies that combining them to remove the redundancy would improve computational efficiency, as suggested in [28]. Nevertheless, extensive computer simulations reveal that the loss due to multiple alignment is negligible whenever we choose up to 75–100 references. Therefore, our recommendation is to use, as a rule of thumb, the threshold 75 in order to achieve the best possible compression ratio and at the same time provide a more complete list of genomic references for further analysis.

### Compression performance analysis

Our chosen comparison quality criteria include the compression ratio (i.e., the ratio of the uncompressed file and the compressed file size), as well as the compression and decompression time, as measured on an affordable general purpose computing platform: Intel Core i5–3470 CPU at 3.2 GHz, with a 16 GB RAM. We present test results for five datasets: ERR321482, SRR359032, ERR532393, SRR1450398, and SRR062462, including metagenomic samples as diverse as a human gut microbiome or a Richmond Mine biofilm sample, retrieved from the NCBI Sequence Read Archive [29]. Additional file 2 contains detailed descriptions of the datasets tested.

**Fig. 2:**
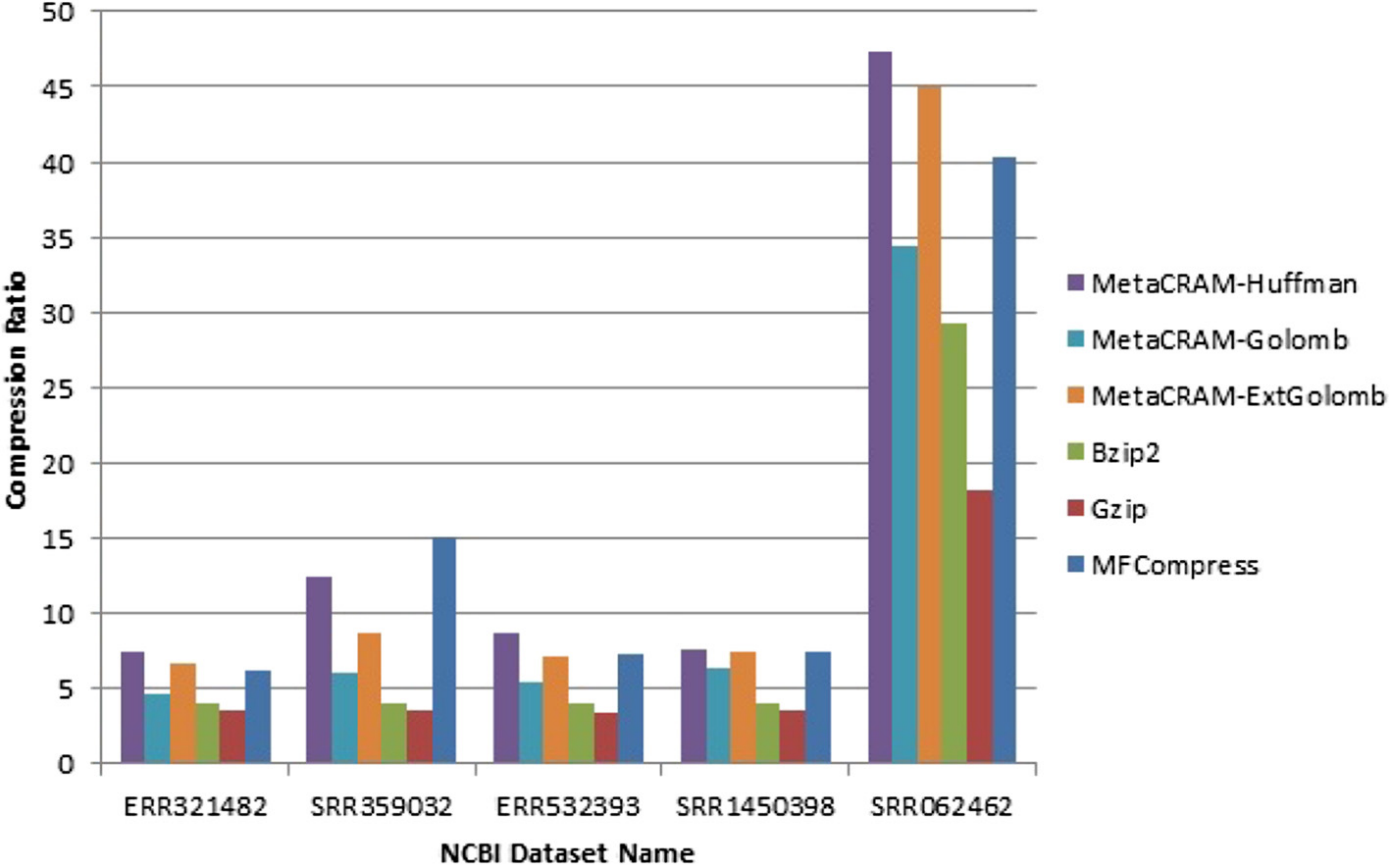
Compression ratio. The compression ratios for all six software suites, indicating the compression ratio

The comparison results of compression ratios among six software suites are given in Table 2 and Fig. 2. The methods compared include three different modes of MetaCRAM, termed Huffman, Golomb and Extended Golomb MetaCRAM. These three techniques differ from each other with respect to the integer compression scheme used. The schemes will be described in detail in the next sections, although we remark that the three methods are chosen to illustrate various compression ratio and decompression time trade-offs.

**Table 2:** Comparison of compression ratios of six software suites

Data	Original (MB)	MCH1 (MB)	MCH2 (MB)	MCG (MB)	MCEG (MB)	Align. %	Qual value (MB)	bzip2 (MB)	gzip (MB)	MFComp (MB)
ERR321482	1429	**191**	186	312	213	29.6	411	362	408	229
SRR359032	3981	319	282	657	458	61.8	2183	998	1133	**263**
ERR532393	8230	**948**	898	1503	1145	46.8	3410	2083	2366	1126
SRR1450398	5399	**703**	697	854	729	7.7	365	1345	1532	726
SRR062462	6478	**137**	135	188	144	2.7	153	222	356	161

For short hand notation, we used“MCH” = MetaCRAM-Huffman, “MCG” = MetaCRAM-Golomb, “MCEG” = MetaCRAM-extended Golomb, “MFComp” = MFCompress. MCH1 is the default option of MetaCRAM with Huffman encoding, and MCH2 is a version of MetaCRAM in which we removed the redundancy in both quality scores and the read IDs. “Align. %” refers to the total alignment rates from the first and second iteration. Minimum compressed file size achievable by the methods are written in bold case letters. Minimum compressed file size achievable by the methods are written in bold case letters

The result indicates that MetaCRAM using Huffman integer encoding method improves compression ratios of the classical gzip algorithm 2–3 fold on average. For example, MetaCRAM reduces the file size of SRR062462 to only 2 % of the original file size. Observe that MetaCRAM also offers additional features that go beyond compression only, such as taxonomy identification and assembly. Users have the options to retrieve the alignment rate, list of reference genomes, contig files, and alignment information in SAM format. This list produced by MetaCRAM may be stored with very small storage overhead and then used for quick identification of files based on their taxonomic content, which allows for selection in the compressive domain. Information regarding gene profiles was not included in the pipeline output, as gene analysis does not directly contribute to the quality of the compression algorithm.

In the listed results, the column named “Qual Value (MB)” provides the estimated size of the quality scores for each file, after alignment to references found by Kraken. In our implementation, we replaced these scores with a single “*” symbol per read and also removed the redundancy in read IDs. The result shows that these two options provide better ratios than the default ratio, as shown in Table 2 column “MCH2”. However, since read IDs may be needed for analysis of some dataset, we also report results for the default “MCH1” mode which does not dispose of ID tags.

**Fig. 3:**
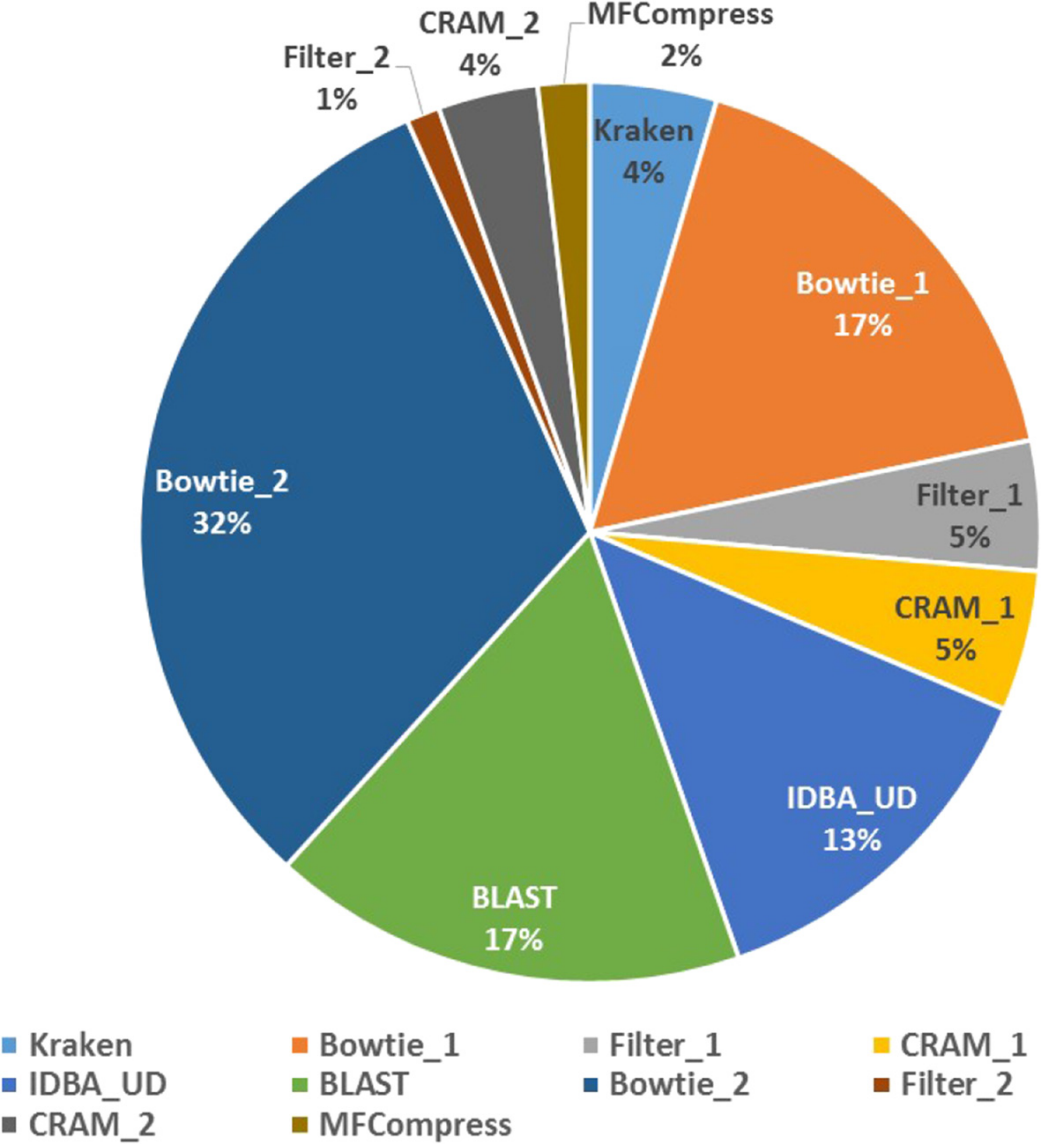
Average Runtime of Each Stage of MetaCRAM. Detailed distribution of the average runtimes of MetaCRAM for all five datasets tested. We used “_1” to indicate the processes executed in the first round, and “_2” to denote the processes executed in the second round

In terms of the processing time shown in Table 3, the MetaCRAM suite is at a clear disadvantage, with processing time 150-fold slower than bzip2 in the worst case. Figure 3 presents the average runtime of each stage for all five datasets tested, and illustrates that assembly, alignment, and BLAST search are computationally demanding, accounting for 62 percentage of the total time. This implies that removing the second and subsequent assembly rounds of MetaCRAM reduces the processing time significantly, at the cost of a smaller compression ratio. Table 4 compares the compression ratios of MetaCRAM with one round and with two rounds of reference discovery, and indicates that removing the assembly, alignment and BLAST steps adds 1–6 MB to the compressed file size. Thus, the user has an option to skip the second round in order to expedite the processing time.

**Table 3:** Comparison of processing (compression) times of six software suites. Times are recorded row by row denoting real, user, and system time in order

Data	Time	MCH	MCG	MCEG	bzip2	gzip	MFComp
ERR321482	real	299 m 20 s	294 m 27 s	274 m 43 s	2 m 2 s	3 m 49 s	2 m 38 s
	user	422 m 21 s	422 m 49 s	402 m 25 s	1 m 56 s	3 m 45 s	4 m 49 s
	sys	12 m 24 s	8 m 48 s	12 m 13 s	0 m 1 s	0 m 1 s	0 m 13 s
SRR359032	real	127 m 34 s	129 m 32 s	128 m 14 s	5 m 36 s	10 m 39 s	8 m 2 s
	user	245 m 53 s	247 m 43 s	253 m 16 s	5 m 19 s	10 m 30 s	13 m 3 s
	sys	8 m 37 s	10 m 1 s	15 m 25 s	0 m 2 s	0 m 2 s	0 m 15 s
ERR532393	real	639 m 55 s	635 m 53 s	641 m 32 s	11 m 28 s	22 m 18 s	17 m 2 s
	user	1061 m 50 s	1069 m 9 s	1090 m 20 s	11 m 4 s	21 m 58 s	28 m 29 s
	sys	73 m 59 s	27 m 59 s	43 m 35 s	0 m 5 s	0 m 5 s	0 m 21 s
SRR1450398	real	440 m 4 s	439 m 42 s	440 m 36 s	7 m 38 s	14 m 39 s	10 m 32 s
	user	66 m 56 s	865 m 38 s	865 m 6 s	7 m 19 s	14 m 24 s	18 m 8 s
	sys	821 m 2 s	23 m 51 s	26 m 5 s	0 m 3 s	0 m 3 s	0 m 18 s
SRR062462	real	217 m 21 s	224 m 32 s	215 m 58 s	2 m 48 s	2 m 6 s	6 m 38 s
	user	254 m 26 s	261 m 19 s	256 m 17 s	2 m 7 s	1 m 18 s	10 m 39 s
	sys	15 m 45 s	16 m 48 s	20 m 14 s	0 m 3 s	0 m 3 s	0 m 16 s

**Table 4:** Comparison of compressed file sizes of MetaCRAM-Huffman using 2 rounds and 1 round

Data	Original (MB)	MCH-2rounds (MB)	Align. %	MCH-1round (MB)	Align. %	gzip (MB)	MFComp (MB)
ERR321482	1429	191	29.6	192	27	408	229
SRR359032	3981	319	61.8	315	57.7	1133	263
ERR532393	8230	948	46.8	952	45.8	2366	1126
SRR1450398	5399	703	7.7	707	7.1	1532	726
SRR062462	6478	137	2.7	143	2.6	356	161

For short hand notation, we used“MCH-2rounds” = MetaCRAM-Huffman with 2 rounds, “MCH-1round” = MetaCRAM-Huffman with 1 round. We also used the shortcut “MFComp” = MFCompress and “Align. %” refers to the percentage of reads aligned during 2 rounds and 1 round, respectively, for MCH-2rounds and MCH-1round

Likewise, Table 5 illustrates that the retrieval time of MetaCRAM is longer than that of bzip2, gzip, and MFCompress, but still highly efficient. In practice, the processing time is not as relevant as the retrieval time, as compression is performed once while retrieval is performed multiple times. For long term archival of data, MetaCRAM is clearly the algorithm of choice since the compression ratio, rather than processing or retrieval time, is the most important quality criteria.

**Table 5:** Comparison of retrieval (decompression) times of six software suites. Times are recorded row by row denoting real, user, and system time in order

Data	Time	MCH	MCG	MCEG	bzip2	gzip	MFComp
ERR321482	real	23 m 17 s	25 m 18 s	24 m 56 s	0 m 57 s	0 m 17 s	2 m 26 s
	user	16 m 17 s	16 m 30 s	17 m 7 s	0 m 45 s	0 m 9 s	4 m 42 s
	sys	9 m 2 s	10 m 42 s	10 m 25 s	0 m 2 s	0 m 1 s	0 m 4 s
SRR359032	real	12 m 16 s	11 m 43 s	13 m 17 s	2 m 37 s	1 m 28 s	7 m 58 s
	user	11 m 59 s	11 m 24 s	12 m 43 s	2 m 8 s	0 m 28 s	15 m 10 s
	sys	2 m 24 s	1 m 42 s	3 m 12 s	0 m 4 s	0 m 2 s	0 m 19 s
ERR532393	real	48 m 19 s	47 m 5 s	55 m 58 s	5 m 25 s	2 m 30 s	15 m 29 s
	user	39 m 59 s	40 m 5 s	43 m 21 s	4 m 23 s	0 m 55 s	29 m 23 s
	sys	15 m 39 s	13 m 25 s	29 m 17 s	0 m 7 s	0 m 5 s	0 m 17 s
SRR1450398	real	28 m 43 s	27 m 54 s	29 m 27 s	3 m 25 s	1 m 54 s	10 m 8 s
	user	29 m 55 s	29 m 47 s	30 m 45 s	2 m 52 s	0 m 37 s	19 m 1 s
	sys	7 m 10 s	5 m 52 s	7 m 4 s	0 m 5 s	0 m 3 s	0 m 26 s
SRR062462	real	23 m 9 s	22 m 55 s	26 m 6 s	1 m 3 s	1 m 19 s	5 m 52
	user	21 m 10 s	21 m 10 s	21 m 58 s	0 m 42 s	0 m 22 s	10 m 31 s
	sys	4 m 49 s	4 m 53 s	10 m 12 s	0 m 4 s	0 m 3 s	0 m 26 s

We also remark on the impact of different integer encoding methods on the compression ratio. Huffman, Golomb, and extended Golomb codes all have their advantages and disadvantages. For the tested datasets, Huffman clearly achieves the best ratio, as it represents the optimal compression method, whereas Golomb and extended Golomb compression improve the real and system time as a result of computation efficiency. However, the parallel implementation of MetaCRAM makes the comparison of processing time of the three methods slightly biased: for example, if we perform compression while performing assembly, compression will take much more time than compressing while running an alignment algorithm. As the processing and retrieval time is not consistent among the three methods, we recommend using Huffman coding for archival storage.

## Discussion

In what follows, we comment on a number of useful properties of the MetaCRAM program, including compatibility, losslessness, partial assembly results and compressive computing.

**Compatibility.** MetaCRAM uses well established and widely tested genomic analysis tools, and it also follows the standard genomic data compression format CRAM, hence making the results of downstream analysis compatible with a current standard for genomic compression.

**Lossless compression principle.** By its very nature, MetaCRAM is a lossless compression scheme as it encodes the differential information between the reference and the metagenomic reads in a 100 % accurate fashion. Nevertheless, we enabled a feature that allow for some partial loss of information, such as the read ID tags. It is left to the discretion of the user to choose suitable options.

**CRAM versus MFCompress.** MFCompress achieves good compression ratios when compressing highly redundant reads. MetaCRAM consistently achieves a rate proportional to the alignment rate because it only encodes the small difference between the reference genome and the read. As more microbial genome become available, MetaCRAM will most likely offer higher compression ratio than other tools in general. Note that only on one data file - SRR359032 - did MFCompress achieve better compression ratios than MetaCRAM, most likely due to the redundancy issues previously mentioned.

**Metagenomic assembly.** Metagenomic assembly is a challenging task, and there is a widely accepted belief that it is frequently impossible to perform meaningful assembly on mixture genomes containing species from related genomes. Nevertheless, we are using assembly mostly as a means for identifications, but at the same time its output provides useful contigs for gene transfer analysis and discovery. In the case that assembly fails on a dataset, we suggest skipping the assembly step so as to trade off computation time with discovery of new reference genomes and contigs.

**Compressive computing.** There has been an effort towards computing in the compressed domain, in order to eliminate the need for persistne compression and decompression time when all one needs to perform is simple alignment [30]. Similarly, MetaCRAM offers easy retrieval and selection based on the list of references stored as an option. For example, suppose we perform MetaCRAM on all available human gut metagenome data. If we want to analyze the datasets with a concentration of *Escherichia coli*, we avoid sacrificing retrieval time by quickly scanning the list of reference files and only retrieving the datasets with *E. coli*.

## Methods

### Pre-Processing

MetaCRAM accepts both unpaired and paired-end reads. If paired-end reads are given as an input to MetaCRAM, then the first preprocessing step is to append the read IDs with a “_1” or a “_2” indicating that the read came from the first or second mate, respectively. Another preprocessing step includes filtering out the quality scores in case that the input file is in FASTQ format. This filtering process allows for using new and emerging quality score compression methods without constantly updating the MetaCRAM platform. Note that the paired end labeling is done automatically, while filtering can be implemented outside the integrated pipeline by the user, based on his/her requirements for quality score lossy or lossless compression goals.

MetaCRAM uses as a default FASTA files that do not contain quality values, in which case the resulting SAM file contains the symbol “I” repeated as many times as the length of the sequence. These symbols amount to about 100 bytes per read, and this overhead increases proportionally to the number of reads (Table 2 of the “Results” Section illustrates the amount of storage space that data quality scores occupy in each dataset, ranging from 153 MB to 3.4 GB). In order to reduce the size of this unnecessary field, MetaCRAM replaces the sequence of “I”s with a single symbol “*”, complying with the standard SAM format. Likewise, read IDs are highly repetitive in nature: for instance, every read ID starts with the data name such as “SRR359032.”, followed by its unique read number. Rather than repeating the data name for every read, we simply store it once, and append it when performing decompression. Both versions of MetaCRAM – one incorporating these two options – and another one without the described features are available to the user. The former version of the methods requires a slightly longer compression and decompression time.

### Taxonomy identification

Given the labeled read sequences of a metagenomic sample, the first step is to identify the mixture of species present in the sample. There are several taxonomy identification methods currently in use: the authors of [31] proposes to use the 16S rRNA regions for bacterial genome identification, MetaPhyler [32] scans for unique markers exceeding length 20 and provides a taxonomy level as specific as the genus. On the other hand, a new taxonomy identification software known as Kraken [15], based on exact alignment of *k*-mers to the database of known species, often outperforms MetaPhyler and other methods both in terms of speed and discovery of true positives, as indicated by our tests.

MetaCRAM employs Kraken as a default tool in the pipeline. Kraken produces an output report which is automatically processed by MetaCRAM. Part of the report contains information about species present in the sample, as well as their abundance. We rank order the species in from the most abundant to the least abundant, where abundance is based on the number of reads identified to match a species in the database. For downstream analysis, MetaCRAM selects the “most relevant” species and uses their genomes as references. The default definition of “most relevant” is the top 75 species, but one has the option to choose a threshold for the abundance value or for the number of references used. As an illustration, Table 1 lists the results of an analysis of the impact of different thresholds on the processing time and the compression ratio.

### Alignment and assembly

After a group of reference genomes is carefully chosen based on the Kraken software output, alignment of reads to the reference genomes is performed. This task is accomplished by using Bowtie2, a standard software tool for ultra-fast alignment of short reads to long genomes. The alignment information is stored in a SAM (Sequence Alignment/Map) file format and subsequently used for compression via reference-based algorithms.

Due to the fact that many species in a metagenome sample have never been sequenced before, some reads will not be aligned to any of the references with high alignment scores, and we collectively refer to them as *unaligned* reads hereafter. In order to discover reference genomes for unaligned reads, we assemble the unaligned reads in a relatively efficient, although often time consuming manner using a metagenomic assembler. Our metagenomic assembler of choice is IDBA-UD [18], given that in our tests it produced the largest number of contigs leading to new reference identification. Alternatives to IDBA-UD include the Ray Meta software [33].

When the reads have high sequencing depth and large overlaps, the *contigs* produced by the assembler may be queried using BLAST to identify the organisms they most likely originated from. The user may choose to BLAST only the top *n* longest contigs, where *n* is a user specified number, but in our analysis we use *all* contigs (which is also the default setting). Subsequently, we align the unaligned reads to the newly found references.

In some rare cases, the assembler may fail depending on the number of species in the metasample and the sequencing depth, in which case one may want to skip the assembly step and compress the unaligned reads in a reference-free manner. For reference-free compression, the software of choice in MetaCRAM is MFCompress [14]. As long as the assembler is successful, one can reduce the volume of unaligned reads by iterating the process of assembly, BLAST, and alignment as illustrated at the top right hand of Fig. 1. All our demonstrations and results are based on two iterations of the described algorithmic loop.

### Distribution of read starting positions

**Fig. 4:**
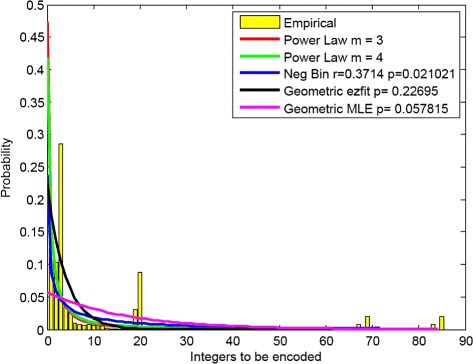
Integer Distribution. Distribution fitting of integers to be encoded, truncated at 90 % of the integer data

We empirically studied the distribution of integers representing the read positions, variation positions, and paired-end offsets in order to choose the most suitable compression method. As an example, the distribution of the starting positions for the reads that aligned to JH603150 (genome of Klebsiella oxytoca) in the dataset SRR359032 is shown in Fig. 4. This distribution was truncated after achieving a 90 % coverage of the data (i.e., after only 10 % of the read start positions exceeded the depicted maximum length). The empirical distribution is shown in yellow, while a fitted power law distributions is plotted and determined according to [22], with $P_{i} = 2^{-\log _{m} i}\frac {1}{2i(m-1)}$, where *i* is the integer to be encoded, and *m* is the divisor in the extended Golomb code. The parameter choose shown is *m*=3 and 4. The negative binomial distribution is fitted using Maximum Likelihood Estimation (MLE), while the Geometric distribution is fitted by two different means: using MLE and ezfit, a MATLAB script which performs an unconstrained nonlinear minimization of the sum of squared residuals with respect to various parameters.

For single reference alignment methods, it was reported that the best fit for the empirical distribution is a geometric distribution or a negative binomial distribution [34]. However, due to sequencing errors and non-uniform distributions of hydrogen bond breakage, the empirical data often deviates from geometric or negative binomial distributions [35]. In addition, for metagenomic samples, there exist multiple references which may have good alignments with reads that did not originally correspond to the genomic sample of the reference. This creates additional changes in the read starting position with respect to the geometric distribution. Moreover, one has to encode not only the read positions but also the variation positions and paired-end offsets, making it difficult to claim any one of the fitted distributions is better than others. This observation is supported by Fig. 4. Since there is no known *efficient* optimal encoding method for a set of integers with negative binomial distributions, and Golomb and extended Golomb encoding are optimal for geometric distributions and power law distributions, respectively, we use these two methods with *m*=3. The parameter *m* is chosen based on extensive experiments, although the user has the freedom to adjust and modify its value.

As the number of unaligned reads that remains after a few iterations of MetaCRAM is relatively small, these reads were compressed using a reference-free tool such as MFCompress [14], which is based on finite-context models. Furthermore, the SAM files produced after running Bowtie2 are converted to the sorted and indexed binary format of a BAM file using SAMtools [36]. Each BAM file is compressed via reference-based compression against its representative to a standard CRAM format. We tested three different modes of the CRAM toolkit [11]: Huffman, Golomb, and Extended Golomb encoding, all of which are described in the next section. Note that the Extended Golomb encoding method is our new addition to the classical CRAM method, as it appears to offer good compromises between compression and decompression speed and compression ratios.

Intrinsically, SAM files contain quality values and unique read IDs for each read, which inevitably account for a large file size: quality values are characters of length as long as the sequence, and read IDs often repeat the name of the dataset. By default, MetaCRAM preserves all quality values and read IDs as designed in CRAM.

#### Compression

Compression in the reference-based mode is accomplished by compressing the starting points of references with respect to the reference genomes and the base differences between the reads and references. As both the starting points and bases belong to a finite integer alphabet, we used three different integer compression methods, briefly described below.

Huffman coding is a prefix-free variable length compression method for known distributions [20] which is information-theoretically optimal [37]. The idea is to encode more frequent symbols with fewer bits than non-frequent ones. For example, given an alphabet *A*=(*a*,*b*,*c*,*d*,*e*) and the corresponding distribution *P*=(0.25,0.25,0.2,0.15,0.15), building a Huffman tree results in the codebook *C*=(00,10,11,010,011). Decoding relies on the Huffman tree constructed during encoding which is stored in an efficient manner, usually ordered according to the frequency of the symbol. Due to the prefix-free property, Huffman coding is uniquely decodable coding and does not require any special marker between words. Two drawbacks of Huffman coding that make it a costly solution for genomic compression are its storage complexity, since we need to record large tree structures for big alphabet size which arise when encoding positions in long sequences and the need to know the underlying distribution *a priori*. Adaptive Huffman coding mitigates the second problem, at the cost of increased computational complexity associated with constructing multiple encoding trees [38]. In order to alleviate computational challenges, we implemented so called *canonical* Huffman encoding, which bypasses the problem of storing a large code tree by sequentially encoding lengths of the codes [39].

Golomb codes are optimal prefix-free codes for countably infinite lists of non-negative integers following a geometric distribution [21]. In Golomb coding, one encodes an integer *n* in two parts, using its quotient *q* and remainder *r* with respect to the divisor *m*. The quotient is encoded in *unary*, while the remainder is encoded via *truncated binary* encoding. Given a list of integers following a geometric distribution with known mean *μ*, the dividend *m* can be optimized so as to reduce code length. In [40], the optimal value of *m* was derived for *m*=2^*k*^, for any integer *k*. The encoding is known as the Golomb-Rice procedure, and it proceeds as follows: first, we let $k^{*}=\textit {max}\Bigg \{0, 1+\left \lfloor \log _{2}\Big (\frac {\log (\phi -1)}{\log \big (\frac {\mu }{\mu +1}\big)}\Big) \right \rfloor \Bigg \}$, where $\phi =\frac {(\sqrt {5}+1)}{2}$. Unary coding represents an integer *i* by *i* ones followed by a single zero. For example, the integer *i*=4 in unary reads as 11110. Truncated binary encoding is a prefix-free code for an alphabet of size *m*, which is more efficient than standard binary encoding. Because the remainder *r* can only take values in {0,1,…, m-1}, according to truncated binary encoding, we assign to the first 2^*k*+1^−*m* symbols codewords of fixed length *k*. The remaining symbols are encoded via codewords of length *k*+1, where *k*=⌊log2(*m*)⌋. For instance, given *n*=7 and *m*=3, we have 7=2×3+1, implying *q*=2 and *r*=1. Encoding 2 in unary gives 110 and 1 in truncated binary reads as 10. Hence, the codeword used to encode the initial integer is the concatenation of the two representations, namely 11010.

Decoding of Golomb encoded codewords is also decoupled into decoding of the quotient and the remainder. Given a codeword, the number of ones before the first zero determines the quotient *q*, while the remaining *k* or *k*+1 bits, represents the remainder *r* according to truncated binary decoding for an alphabet of size *m*. The integer *n* is obtained as *n*=*q*×*m*+*r*.

Golomb encoding has one advantages over Huffman coding in so far that it is computationally efficient (as it only requires division operations). One does not need to the distribution *a priori*, although there are clearly no guarantees that Golomb coding for an unknown distribution will be even near-optimal: Golomb encoding is optimal only for integers following a geometric distribution.

An extension of Golomb encoding, termed *extended* Golomb [22] coding, is an iterative method for encoding non-negative integers following a power law distribution. One divides an integer *n* by *m* until the quotient becomes 0, and then encodes the number of iterations *M* in unary, and an array of remainders *r* according to an encoding table. This method has an advantage over Golomb coding when encoding large integers, such is the case for read position compression. As an example, consider the integer *n*=1000: with *m*=2, Golomb coding would produce *q*=500 and *r*=0, and unary encoding of 500 requires 501 bits. With extended Golomb coding, the number of iterations equals *M*=9 and encoding requires only 10 bits. As an illustration, let us encode *n*=7 given *m*=3. In the first iteration, 7=2×3+1, so *r*_1_=1 is encoded as 10, and *q*_1_=2. Since the quotient is not 0, we iterate the process: 2=0×3+2 implies *r*_2_=2, which is encoded as 1, and *q*_2_=0. Because the quotient is at this step 0, we encode *M*=2 as 110 and *r*=*r*_2_*r*_1_=110, and our codeword is 110110.

The decoding of extended Golomb code is also performed in *M* iterations. Since we have a remainder stored at each iteration and the last quotient equals *q*_*M*_=0, it is possible to reconstruct the original integer. Similar to Golomb coding, extended Golomb encoding is computationally efficient, but optimal only for integers with power law distribution.

There are various other methods for integer encoding, such as Elias Gamma and Delta Encoding [41], which are not pursued in this paper due to the fact that they do not appear to offer good performance for the empirical distributions observed in our read position encoding experiments.

### Products

The compressed unaligned reads, CRAM files, list of reference genomes (optional), alignment rate (optional), contig files (optional) are all packaged into an archive. The resulting archive can be stored in a distributed manner and when desired, the reads can be losslessly reconstructed via the CRAM toolkit. Additional file 3 contains software instructions, and detailed descriptions of created files and folders by MetaCRAM processing are available in Additional file 4.

### Decompression

Lossless reconstruction of the reads from the compressed archive is done in two steps. For those reads with known references in CRAM format, decompression is performed with an appropriate integer decompression algorithm. When the files are converted back into the SAM format, we retrieve only the two necessary fields for FASTA format, i.e., the read IDs and the sequences printed in separate lines. Unaligned reads are decompressed separately, through the decoding methods used in MFCompress.

### Post-processing

The two parts of reads are now combined into one file, and they are sorted by the read IDs in an ascending order. If the reads were paired-end, they are separated into two files according to the mate “flag” assigned in the processing step.

### Effects of parallelization

One key innovation in the implementation of MetaCRAM is parallelization of the process, which was inspired by parallel *single genome* assembly used in TIGER [42]. Given that metagenomic assembly is computationally highly demanding, and in order to fully utilize the computing power of a standard desktop, MetaCRAM performs meta assembly of unaligned reads and compression of aligned reads in parallel. As shown in Table 6, parallelization improves real, user, and system time by 23–40 %.

**Table 6:** Processing time improvements for two rounds of MetaCRAM on the SRR359032 dataset (5.4 GB, without removing redundancy in description lines) resulting from parallelization of assembly and compression

Time	Without parallelization	With parallelization	Reduction (%)
Real	235 m 40 s	170 m 4 s	27.7
User	449 m 40 s	346 m 33 s	22.9
System	14 m 13 s	8 m 45 s	40.1

## Availability of supporting data

The datasets supporting the results of this article are available in the National Center for Biotechnology Information Sequence Read Archive repository, under accession numbers ERR321482 (http://www.ncbi.nlm.nih.gov/sra/ERX294615), SRR359032 (http://www.ncbi.nlm.nih.gov/sra/SRX103579), ERR532393 (http://www.ncbi.nlm.nih.gov/sra/ERX497596), SRR1450398 (http://www.ncbi.nlm.nih.gov/sra/SRX621521), SRR062462 (http://www.ncbi.nlm.nih.gov/sra/SRX024927).

## Conclusions

We introduced MetaCRAM, the first parallel architecture for reference-based, lossless compression of metagenomic data. The compression scheme is compatible with standard CRAM formats and offers significantly improved compression ratios compared to the existing software suites, compressing file to 2-13 percent of the original size. Furthermore, it provides the user with taxonomy and assembly information, allowing for fast selection of relevant files in the compressed domain. Thus, MetaCRAM may represent an important processing platform for large metagenomic files.

## Additional files

Additional file 1
**Comparison between Kraken and MetaPhyler.** We compare two taxonomy identification tools and decide that Kraken outperforms MetaPhyler in terms of both speed and accuracy. Additional file 4 contains a table of randomly selected set of 15 species used for the comparison. (PDF 11 kb)

Additional file 2
**Datasets used for testing MetaCRAM.** This file has detailed descriptions of the 5 datasets used for testing MetaCRAM, with unique ID specified in the NCBI SRA and the type of library used. (PDF 13 kb)

Additional file 3
**Software instruction.** This file includes specific commands to compress or decompress MetaCRAM, including options available. It also contains default software commands used for each step of MetaCRAM pipeline. (PDF 117 kb)

Additional file 4
**Outcome of MetaCRAM.** Additional file 2 illustrates detailed outcome of MetaCRAM, such as files and folders produced after compression and decompression, and an example of console output. (PDF 82 kb)
