# Supplementary material for: MetaCRAM: an integrated pipeline for metagenomic taxonomy identification and compression
Source: BMC Bioinformatics. 2016 Feb 19;17:94. doi: 10.1186/s12859-016-0932-x (PMC4759986; doi:10.1186/s12859-016-0932-x)
Supplement: Additional file 1 — Comparison between Kraken and MetaPhyler. We compare two taxonomy identification tools and decide that Kraken outperforms MetaPhyler in terms of both speed and accuracy. Additional file 4 contains a table of randomly selected set of 15 species used for the comparison. (PDF 11 kb) [file 12859_2016_932_MOESM1_ESM.pdf]

MetaCRAM Additional File 1

Commands to run MetaCRAM

Compression

time perl MetaCram.pl --<compress, decompress> --output <output directory> --paired <path to reads> --<exGolomb, huffman, golomb>

Example:
[shared3]$ time perl MetaCram.pl --compress --output /shared3/MetaCRAM_SRR359032_Huffman --paired /shared3/SRR359032_1.fasta /shared3/SRR359032_2.fasta --huffman &>MetaCramLOG_SRR359032_Huffman.txt

Decompression:
time perl decompressor.pl --input <path to folder containing the Round1 and Round2 folders>

Example:
[shared3]$ time perl decompressor.pl --input /shared3/MetaCRAM_processedSRR359032_Huffman/MetaCRAM &>decompressorLogSRR359032_Huffman.txt

(*--paired is optional)
(*<> indicates a choice)
(* to log, append “&> <log file>”)
(* “time” command to get real, user, system run time)

Software commands/options used in MetaCRAM

Kraken

Input: fasta/fastq read file
Output: taxonomy identification
Command: kraken --db <database_location> <fasta file> --threads <# threads>
Database: minikraken_20140330

Bowtie2

Indexing
Input: reference genomes
Output: index of reference genomes
Command: bowtie2-build -f *<list of .fna reference genome files> <Output: index result file name>*
Aligning
Input: index of reference genomes, reads to be aligned

Output: SAM file with reads aligned to reference genomes
Command: bowtie2 --reorder --threads 4 --mm -x <*index file name*> -1 <*read1 file name*> -2 <*read2 file name*> -S <*Output: SAM result file name*> -f --no-hd --no-sq

IDBA_UD

Input: read file
Output: contig files
Command: idba_ud --num_threads 4 -r <*read file name*> -o <*output directory desired*>

BLAST

Input: contig
Output: BLAST result (alignment to species)
Command: blastn -db nt -query <query sequence file> -out <output file name desired> -num_threads 4 -max_target_seqs 1 -outfmt “6 qseqid sseqid sgi sacc stitle evalue”

Retrieving subject’s sequence from blast database (command line)

Input: a file containing a list of sequence IDs (accession number and GI)
Output: a fasta file with all sequences of the input file
Command: export BLASTDB=/opt/ncbi-blast-2.2.29+/db; blastdbcmd -db nt -entry_batch <input file name> -out <output file name desired> -outfmt %f

CRAM

Input: split sam file containing records pertaining to that ID (NC_013361) and the corresponding fasta sequence with ending .fa
Output: cram file
Commands: samtools view -bT NC_013361.fa NC_013361.sam | samtools sort - Sorted_NC_013361
To compress: java -jar ../cramtools-2.1.jar cram --reference-fasta-file NC_013361.fa --input-bam-file Sorted_NC_013361.bam --output-cram-file NC_013361.cram
To decompress: java -jar ../cramtools-2.1.jar bam --input-cram-file NC_013361.cram --reference-fasta-file NC_013361.fa --print-sam-header > test.sam

MFCompress

Input: fasta file
Output: compressed fasta file .mfc
Command: ./MFCompressC <filename.fa>
