# Supplementary material for: MetaCRAM: an integrated pipeline for metagenomic taxonomy identification and compression
Source: BMC Bioinformatics. 2016 Feb 19;17:94. doi: 10.1186/s12859-016-0932-x (PMC4759986; doi:10.1186/s12859-016-0932-x)
Supplement: Additional file 2 — Datasets used for testing MetaCRAM. This file has detailed descriptions of the 5 datasets used for testing MetaCRAM, with unique ID specified in the NCBI SRA and the type of library used. (PDF 13 kb) [file 12859_2016_932_MOESM2_ESM.pdf]

MetaCRAM Additional File 2

Folders and files produced after compression

assembled 🗁
→round1 🗁
output of IDBA_UD: contig files
blastOUT 🗁
→round1 🗁
→fnaFiles 🗁
reference files after running BLAST on contigs
blastOUT
out.fna (concatenated file of references)
blastOUT_parsed.txt (name of reference files)
bowtieOutput 🗁
→samSplit 🗁
SAM files split according to the references the reads were aligned to.
bowtie outputs and indices
cramOutput 🗁
→round1 🗁
.bam, .cram, .fa, .fa.fai files for round 1
→round2 🗁
.bam, .cram, .fa, .fa.fai files for round 1
krakenOutput 🗁
KrakenOutput
KrakenOutputReport
listOfFnaFiles
Round1 🗁
listOfFnaFiles
.cram files
Round2 🗁
unaligned2.mfc
blastOUT_parsed.txt
.cram files
SAMFilter 🗁
→round1 🗁
unaligned1
unpaired
unaligned1_1.fna
unaligned1_2.fna
→round2 🗁
unaligned2

unpaired
unaligned2_1.fna
unaligned2_2.fna

indicates a folder rather than a file.
Example given above is for two iterations. More iteration will have more folders named round3, round4, etc. and files accordingly.
Round1 and Round2 are the only folders needed for a lossless decompression.

Files produced after decompression

OUT (original FASTA file containing reads, or in case of paired-end reads, the first pair)
OUT2 (in case of paired-end reads, the second pair)
sortedOUT (sorted version of OUT, according to the read IDs)
sortedOUT2 (sorted version of OUT2, according to the read IDs)

Example of console output of MetaCRAM

```
[minji@algorithm shared3]$ perl MetaCram.pl --compress --output /shared3/
MetaCRAM_SRR359032_Huffman --paired /shared3/SRR359032_1.fasta /shared3
/SRR359032_2.fasta --huffman
Making necessary directories. Done.
17345097 sequences (3486.36 Mbp) processed in 667.124s (1560.0 Kseq/m, 313.56 Mbp/m).
 10127714 sequences classified (58.39%)
 7217383 sequences unclassified (41.61%)
Done.
Filtering Kraken output.
Gathering .fna files
Using variation to find Sphingobium_sp_SYK-6
Using genus to find Sphingobium_sp_SYK-6
Using variation to find Rhodanobacter_denitrificans
Using variation to find Paenibacillus_sp_Y412MC10
Using variation to find Methylocystis_sp_SC2
Using variation to find Geobacter_sp_M18
Filter complete.
Round 1
Running bowtie2-build. Building a SMALL index
Done.
Running bowtie2. 17345097 reads; of these:
 17345097 (100.00%) were paired; of these:
    7909091 (45.60%) aligned concordantly 0 times
    9334717 (53.82%) aligned concordantly exactly 1 time
    101289 (0.58%) aligned concordantly >1 times
    ----
    7909091 pairs aligned concordantly 0 times; of these:
     46710 (0.59%) aligned discordantly 1 time
    ----
    7862381 pairs aligned 0 times concordantly or discordantly; of these:
     15724762 mates make up the pairs; of these:
```

```
      14668312 (93.28%) aligned 0 times
      1016579 (6.46%) aligned exactly 1 time
      39871 (0.25%) aligned >1 times
57.72% overall alignment rate
Done.
Separating SAM file into individual species' SAM file.
Done.
Running CRAM.
Opening SAM file. Done.
Making unaligned file. Done.
Making unpaired file. Done.
Filtering SAM file. Done.
Splitting unaligned into 2 files. Done.
Running IDBA_UD. [bam_sort_core] merging from 9 files...
Done.
BLASTing.
Done.
Filtering BLAST output.
Retrieving fna sequence from BLAST database. Done.
Parsing BLAST fna sequences. Done.
Done.
Round 2
Running bowtie2-build. Building a SMALL index
Done.
Running bowtie2. 6810363 reads; of these:
 6810363 (100.00%) were paired; of these:
    6241196 (91.64%) aligned concordantly 0 times
    484436 (7.11%) aligned concordantly exactly 1 time
    84731 (1.24%) aligned concordantly >1 times
    ----
    6241196 pairs aligned concordantly 0 times; of these:
     7482 (0.12%) aligned discordantly 1 time
    ----
    6233714 pairs aligned 0 times concordantly or discordantly; of these:
     12467428 mates make up the pairs; of these:
      12299670 (98.65%) aligned 0 times
      139320 (1.12%) aligned exactly 1 time
      28438 (0.23%) aligned >1 times
9.70% overall alignment rate
Done.
Separating SAM file into individual species' SAM file.
Done.
Running CRAM.
Reading start positions. Opening SAM file. Done.
Making unaligned file. Done.
Making unpaired file. Done.
Filtering SAM file. Done.
Done.
Splitting unaligned into 2 files. Done.
Running MFCompress.
Done.
```
