# Supplementary material for: MetaCRAM: an integrated pipeline for metagenomic taxonomy identification and compression
Source: BMC Bioinformatics. 2016 Feb 19;17:94. doi: 10.1186/s12859-016-0932-x (PMC4759986; doi:10.1186/s12859-016-0932-x)
Supplement: Additional file 3 — Software instruction. This file includes specific commands to compress or decompress MetaCRAM, including options available. It also contains default software commands used for each step of MetaCRAM pipeline. (PDF 117 kb) [file 12859_2016_932_MOESM3_ESM.pdf]

MetaCRAM Additional File 3

Description of the datasets used for testing MetaCRAM

1) ERR321482: Population Genomics of human gut metagenome
   1 ILLUMINA (Illumina HiSeq 2000) run: 6.8M spots, 1.2G bases, 720.8Mb downloads
   Abstract
   We are facing a global metabolic health crisis provoked by an obesity epidemic. Here we report the human gut microbial composition in a population sample of 123 non-obese and 169 obese Danish individuals. We find two groups of individuals that differ by the number of gut microbial genes and thus gut bacterial richness. They harbour known and previously unknown bacterial species at different proportions; individuals with a low bacterial richness (23% of the population) are characterized by more marked overall adiposity, insulin resistance and dyslipidaemia and a more pronounced inflammatory phenotype when compared with high bacterial richness individuals. The obese individuals among the former also gain more weight over time. Only a few bacterial species are sufficient to distinguish between individuals with high and low bacterial richness, and even between lean and obese. Our classifications based on variation in the gut microbiome identify subsets of individuals in the general white adult population who may be at increased risk of progressing to adiposity-associated co-morbidities.
   Library:
   Name: 101205
   Instrument: Illumina HiSeq 2000
   Strategy: WGS
   Source: METAGENOMIC
   Selection: other
   Layout: PAIRED
2) SRR359032: Illumina sequencing of community genomic DNA from Richmond Mine Abmuck location biofilm August 2007
   1 ILLUMINA (Illumina HiSeq 2000) run: 17.3M spots, 3.5G bases, 2.3Gb downloads
   Abstract
   Richmond Mine acid mine drainage biofilm samples datasets (2006-2010). This project describes a population genomic study focused on determining the evolutionary rates in natural populations, and to correlate human disturbance to major evolutionary divergence events. Biofilm samples from acid mine drainage were taken in the period 2006-2010 at four locations in the Richmond Mine (Iron Mt., CA). Community genomic DNA was extracted and sequenced by either 454 or Illumina sequencing.

Library:
Name: ILLAMDABmuck0807
Instrument: Illumina HiSeq 2000
Strategy: WGS
Source: METAGENOMIC
Selection: RANDOM
Layout: PAIRED

3) ERR532393: Population Genomics of metagenome sequence
1 ILLUMINA (Illumina HiSeq 2000) run: 35.8M spots, 7.2G bases, 4.9Gb downloads
Abstract:
Liver cirrhosis occurs as a consequence of many chronic liver diseases that are prevalent worldwide. Previous studies have shown an association between the gut microbiota and liver complications such as cirrhosis and other liver injuries. We therefore undertook a whole gut microbiome wide association study of stool samples from 98 liver cirrhosis patients and 83 healthy controls to characterise the faecal microbial communities and their functional composition. In total, we generated 860 Gb of high-quality sequence data and built a reference gene set for the liver cirrhosis cohort containing 2.69 million genes, 36.1% of which was not covered by previously published gene catalogues.
Library:
Name: unspecified
Instrument: Illumina HiSeq 2000
Strategy: WGS
Source: METAGENOMIC
Selection: size fractionation
Layout: PAIRED

4) SRR1450398: Biocathode Biofilm Metagenome
1 ILLUMINA (Illumina HiSeq 2000) run: 23.3M spots, 4.7G bases, 3Gb downloads
Abstract:
This study integrated both metagenomics and metaproteomics of the biocathode biofilm in a microbial fuel cell. Metagenomic sequences deposited here reflect DNA isolated from graphite cathode. Metaproteome samples were also isolated from the same material. Metaproteome data has been submitted to the PRIDE proteomics data repository as described in the accompanying manuscript.
Library:
Name: X41
Instrument: Illumina HiSeq 2000

Strategy: WGS
Source: METAGENOMIC
Selection: other
Layout: PAIRED

5) SRR062462: Metagenomics of human microbiome
2 ILLUMINA (Illumina Genome Analyzer II) runs: 56.3M spots, 11.3G bases, 6.3Gb downloads
Abstract:
The HMP metagenomes production phase represents the shotgun sequencing of metagenomic DNA extracted from samples taken from multiple body sites across hundreds of human subjects. Coupled with the other data generated during the HMP project, these results will provide insights into the genes and pathways present in the human microbiome. We are learning more and more about the ways that human health is influenced by the complex and dynamic communities of microbes (the human microbiota) present on and within our bodies. Disruptions in these communities may trigger or influence the course of various disease states. Preliminary studies have shown this to be the case for certain diseases. The Human Microbiome Project expands on these studies to better understand, prevent and treat many human diseases.
Library:
Name: 2857254302
Instrument: Illumina Genome Analyzer II
Strategy: WGS
Source: GENOMIC
Selection: RANDOM
Layout: PAIRED
