# Supplementary material for: MetaCRAM: an integrated pipeline for metagenomic taxonomy identification and compression
Source: BMC Bioinformatics. 2016 Feb 19;17:94. doi: 10.1186/s12859-016-0932-x (PMC4759986; doi:10.1186/s12859-016-0932-x)
Supplement: Additional file 4 — Outcome of MetaCRAM. Additional file 2 illustrates detailed outcome of MetaCRAM, such as files and folders produced after compression and decompression, and an example of console output. (PDF 82 kb) [file 12859_2016_932_MOESM4_ESM.pdf]

MetaCRAM Additional File 4

Comparison between Kraken and MetaPhyler

We compared Kraken with MetaPhyler in terms of computation time and identification accuracy on synthetic data. The following is a list of randomly selected set of 15 species and their NC file number, and the paired-end read files were generated by MetaSim with 1% error.

Table 1. A randomly selected set of 15 species and their NC file number used for comparing different taxonomy identification tools.

#	NC file number	Species
1	NC_016894	Acetobacterium woodii
2	NC_016795	Brucella abortus
3	NC_017307	Corynebacterium pseudotuberculosis
4	NC_013743	Haloterrigena turkmenica
5	NC_006512	Idiomarina loihiensis
6	NC_018106	Klebsiella oxytoca
7	NC_008526	Lactobacillus casei
8	NC_003210	Listeria monocytogenes
9	NC_007626	Magnetospirillum magneticum
10	NC_009975	Methanococcus maripaludis
11	NC_014391	Micromonospora aurantiaca
12	NC_018413	Mycoplasma gallisepticum
13	NC_013960	Nitrosococcus halophilus
14	NC_007948	Polaromonas JS666
15	NC_019670	Pseudomonas putida
